# Age-dependent resistance to Porcine reproductive and respiratory syndrome virus replication in swine

**DOI:** 10.1186/1743-422X-6-177

**Published:** 2009-10-27

**Authors:** Kelly L Klinge, Eric M Vaughn, Michael B Roof, Elida M Bautista, Michael P Murtaugh

**Affiliations:** 1 Boehringer Ingelheim Vetmedica Inc, 2501 North Loop Drive, Suite 1000, Ames, IA, 50014, USA; 2 Boehringer Ingelheim Vetmedica, 2621 N Belt Highway, St Joseph, MO, 64506, USA; 3 Department of Veterinary and Biomedical Sciences, University of Minnesota, 1971 Commonwealth Avenue, St Paul, MN, 55108, USA

## Abstract

**Background:**

Porcine reproductive and respiratory syndrome virus (PRRSV) causes a prolonged, economically devastating infection in pigs, and immune resistance to infection appears variable. Since the porcine adaptive immune system is not fully competent at birth, we hypothesized that age influences the dynamics of PRRSV infection. Thus, young piglets, growing 16-20-week-old finisher pigs, and mature third parity sows were infected with virulent or attenuated PRRSV, and the dynamics of viral infection, disease, and immune response were monitored over time.

**Results:**

Virulent PRRSV infection and disease were markedly more severe and prolonged in young piglets than in finishers or sows. Attenuated PRRSV in piglets also produced a prolonged viremia that was delayed and reduced in magnitude, and in finishers and sows, about half the animals showed no viremia. Despite marked differences in infection, antibody responses were observed in all animals irrespective of age, with older pigs tending to seroconvert sooner and achieve higher antibody levels than 3-week-old animals. Interferon γ (IFN γ) secreting peripheral blood mononuclear cells were more abundant in sows but not specifically increased by PRRSV infection in any age group, and interleukin-10 (IL-10) levels in blood were not correlated with PRRSV infection status.

**Conclusion:**

These findings show that animal age, perhaps due to increased innate immune resistance, strongly influences the outcome of acute PRRSV infection, whereas an antibody response is triggered at a low threshold of infection that is independent of age. Prolonged infection was not due to IL-10-mediated immunosuppression, and PRRSV did not elicit a specific IFN γ response, especially in non-adult animals. Equivalent antibody responses were elicited in response to virulent and attenuated viruses, indicating that the antigenic mass necessary for an immune response is produced at a low level of infection, and is not predicted by viremic status. Thus, viral replication was occurring in lung or lymphoid tissues even though viremia was not always observed.

## Background

Porcine reproductive and respiratory syndrome virus (PRRSV) is a member of the enveloped, positive-sense, single-stranded RNA virus family *Arteriviridae *[1]. Since its emergence in the late 1980's, PRRSV has become the most important pathogen in the swine industry [2,3].

PRRSV is a dynamic agent that evolves by mutation and recombination [4,5]. Type II isolates, which originated in North America with strain VR2332 as the prototype, show extensive variation in virulence and pathogenesis [6-8]. Variation in virulence and level of immune response is associated with peak levels and duration of viremia [8]. Host animal age also influences the infectious process. Higher levels of viremia and viral excretion were observed in young versus older pigs [9,10], and age-dependent variation in disease severity also was observed in young pigs [11].

These observations suggest that the course of PRRSV infection and the corresponding anti-PRRSV immune response might involve variation in both viral strain and host age. Therefore, we evaluated the virological and immunological responses of young, growing, and mature swine to infection with virulent or attenuated forms of the same Type II PRRSV to elucidate age-related mechanisms of resistance to infection.

We observed marked differences in peak and duration of viremia that were dependent on animal age and viral virulence. A pronounced humoral immune response to PRRSV was consistently induced irrespective of animal age, but cell-mediated immunity was more robust in mature sows. These findings suggest that animal age, or physiological maturity, perhaps due to increased innate immune resistance, strongly influences the outcome of infection, whereas an effective adaptive immune response is triggered at a low threshold of infection that is independent of age.

## Results

### Clinical signs and disease

Pigs in all age groups that were infected with virulent JA142 PRRSV showed clinical signs of PRRS, including coughing, which were slightly more severe in piglets (Table 1). Clinical signs were not evident in the attenuated ATP PRRSV-exposed and negative control animals. Ten pigs inoculated with virulent JA142 PRRSV died during the study. Causes of death varied, but only one, a finisher, was attributed to PRRS-related complications. One untreated piglet also died from a bacterial infection.

**Table 1:** Effect of PRRSV on clinical signs, clinical scores, and weight gain in pigs of various ages.

	**Treatment Group**		
	**Control**	**ATP**	**JA142**
Clinical signs and scores			
Piglet	Normal	Normal	Mild cough, days 7-63 Range 3.0-6.3 (peak on day 16)
Finisher	Normal	Normal	Mild, sporadic cough Range 3.0-4.0 (peak on day 22)
Sow	Normal	Normal	Mild cough, days 12-63 Range 3.0-4.3 (peak on day 12)
Weight gain 0-28 d			
Piglet	0.9	0.9	0.4*
Finisher	2.1	2.3	0.9*
Weight gain 28-63 d			
Piglet	1.7	1.3	1.1*
Finisher	1.8	1.8	2.0

* Lower average daily weight gain (ADWG), than other groups in the same row, two-sample t-test, p < 0.05.

Piglets, which were the fastest growing group, showed a significantly reduced average daily weight gain (ADWG) at 28 and 63 days when infected with virulent PRRSV (Table 1). By contrast, inoculation with attenuated ATP PRRSV had no effect on ADWG in piglets. Twenty-week-old pigs only showed reduced weight gain at 28 days when infected with virulent JA142 PRRSV (Table 1). There was no effect of PRRSV on weight gain in mature sows.

### Characteristics of infection

**Figure 1:**
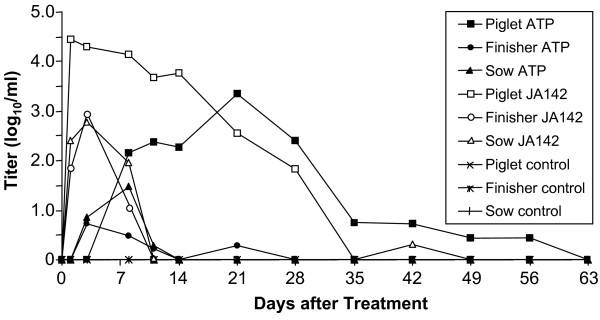
**Effect of pig age and viral strain on PRRSV viremia**. Data are group mean titers determined by limiting dilution culture on MA-104 cells.

Twenty-six of 30 animals in all age groups receiving virulent, JA142 PRRSV were viremic by day 1 and 100% were viremic on day 3 (Table 2). Viremia peaked on day 3 in finishers and sows with mean group titers of about 3.0 log_10 _TCID_50_/mL (Figure 1). All animals in these groups cleared virus below the level of TCID_50 _detection (≤10^1^) by day 11 and, with one exception, remained negative to the end of the study. The exception, a sow, showed a low titer one time, on day 42. By contrast, viremia in piglets peaked on day 1 at a significantly higher titer of 4.5 log_10 _TCID_50_/mL. All piglets were viremic through 14 days of infection, and 6 of 7 were viremic at 21 days (Table 2). All piglets were negative at day 35.

**Table 2:** Proportion of PRRSV-positive animals by viral isolation on MA104 cells.

	Proportion of viremic animals at the indicated days of infection*													
**Treatment**	**Pig age**	**0**	**1**	**3**	**8**	**11**	**14**	**21**	**28**	**35**	**42**	**49**	**56**	**63**
ATP	Piglet	0/10	0/10	0/10	**7/10**	**8/10**	**8/10**	**9/10**	**8/10**	**8/10**	**3/10**	**3/10**	**2/10**	0/10
	Finisher	0/10	0/10	**3/10**	**2/10**	**1/10**	0/10	**2/10**	0/10	0/10	0/10	0/10	0/10	0/10
	Adult	0/10	0/10	**4/10**	**5/10**	**4/10**	0/10	0/10	0/10	0/10	0/10	0/10	0/10	0/10
JA142	Piglet	0/10	**9/9**	**9/9**	**9/9**	**9/9**	**9/9**	**6/7**	**4/7**	0/6	0/6	0/6	0/6	0/6
	Finisher	0/10	**8/10**	**10/10**	**4/9**	0/9	0/9	0/9	0/8	0/8	0/8	0/8	0/8	0/8
	Adult	0/10	**9/10**	**10/10**	**7/10**	0/10	0/9	0/9	0/8	0/8	0/8	0/8	0/7	0/6
None	Piglet	0/10	0/10	0/10	0/10	0/10	0/10	0/10	0/10	0/10	0/10	0/10	0/10	0/10
	Finisher	0/10	0/10	0/10	0/10	0/10	0/10	0/10	0/10	0/10	0/10	0/10	0/10	0/10
	Adult	0/10	0/10	0/10	0/10	0/10	0/10	0/10	0/10	0/10	0/10	0/10	0/10	0/10

* All groups with at least one positive animal are shown in bold.

The animals exposed to ATP PRRSV showed a substantially different pattern of viremia. The highest viremic load was observed in piglets, as was observed with virulent JA142, but virus was not detected until day 8, when 7 of 10 animals were positive. Two animals remained negative until day 21 (Table 2). Peak viremia, at 3.3 log_10 _TCID_50_/mL, occurred on day 21 and viral load declined slowly and variably. Seven of 10 piglets cleared the ATP PRRSV by day 42, but sporadic low level positives were observed for the duration of the study. By contrast, in growing finishers and sows, only 3 to 4 of 10 animals had detectable levels of viremia on day 3 (Table 2). Peak mean group titers were reached on day 3 in finishers (0.73 log_10 _TCID_50_/mL) and on day 8 in sows (1.49 log_10 _TCID_50_/mL), but variation in viremia among animals was substantial. Five finishers and four sows did not show viremia during the entire study. Viremia was not observed in finishers or sows after 11 days except for 2 finishers that were viremic on day 21. All 30 non-challenged control animals remained PRRSV-negative for the duration of the 63 day study.

Viremia also was determined by qRT-PCR; the results were significantly correlated with viral isolation on MA-104 cells. Spearman correlation coefficients ranged from 0.6 to 0.8 and all were significant (p < 0.05). The qRT-PCR findings confirmed the TCID_50_/mL results obtained by growth on MA-104 cells, indicating that both the virulent and attenuated strains grew equivalently in cell culture. In young piglets infected with virulent JA142, viremia was high on day 1 and remained high until day 28, after which it declined substantially. In finishers and sows exposed to JA142, viremia was high from day 1 to day 14, then declined dramatically. Attenuated ATP PRRSV elicited similar kinetics in young, growing and mature pigs; a gradual increase until day 21 followed by a gradual decline in piglets, and brief, low-level viremia in finishers and sows (data not shown).

Except for day 1, when the copies/mL of viral RNA were significantly different among all three age groups, JA142-infected piglets had significantly higher levels of viremia than both finishers and sows, which were equivalent (t-test, p < 0.05). ATP PRRSV-treated piglets also had significantly higher levels of viremia by qRT-PCR than finishers or sows on days 14 through 42, day 56, and day 63.

### Characteristics of the immune response

**Figure 2:**
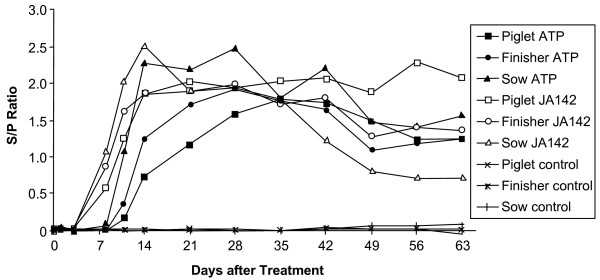
**Effect of age and viral strain on anti-PRRSV antibody response**. Data are group mean sample-to-positive (S/P) ratios determined by HerdChek^® ^PRRS 2XR ELISA.

All pigs showed the same serological response to acute infection with virulent JA142 regardless of age. As shown in Figure 2, all group means were positive at day 8 and peaked at 14 to 21 days, as determined by HerdChek^® ^PRRS 2XR ELISA. All groups maintained a positive sample-to-positive (S/P) ratio for the duration of the study (Fig. 2). Sows showed a substantial decline in S/P ratio after 35 days, while piglets showed the highest average S/P ratio from 49 to 63 days after infection.

Animals inoculated with attenuated ATP PRRSV seroconverted at 10 to 14 days, with sows responding on average more rapidly than finishers and piglets. The response of sows to attenuated ATP PRRSV peaked at 14 days, whereas finishers showed increased mean S/P ratios until day 28 and piglets showed a gradual increase in S/P ratio up to 35 days. Thereafter, animals appeared to equalize and then maintain comparable S/P ratios for the duration of the study.

**Figure 3:**
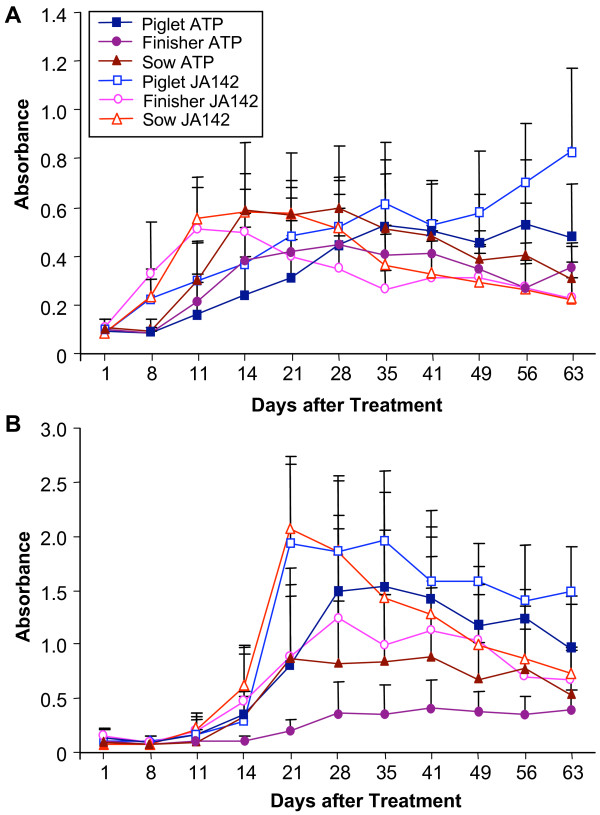
**Effect of age and viral strain on antibody responses to specific structural and nonstructural PRRSV proteins**. (A) Anti-nucleocapsid antibodies. (B) Anti-nonstructural protein 2 antibodies. Data are ELISA absorbance values (mean ± 1 standard deviation) of 10 animals per group. Treatment group legend is shown in panel A.

In Figure 2, the antibody response of piglets to attenuated PRRSV appeared to be delayed. To further investigate this possibility, we examined antibody responses to two specific viral polypeptides. The kinetics of anti-N responses were essentially the same among all ages as determined by HerdChek^® ^PRRS 2XR ELISA, including the declining response of sows infected with virulent PRRSV, and the increasing level of anti-N antibodies from days 49 to 63 in piglets infected with virulent PRRSV (Fig. 3A). The response of piglets to attenuated PRRSV was not significantly different from that of other age groups. Antibody responses to a second viral antigen, an antigenic polypeptide fragment of nonstructural protein 2 (nsp2Hp), showed another pattern of reactivity. Here, virulent JA142 PRRSV elicited antibody responses that peaked at 21 to 28 days in all three age groups, then declined slightly in piglets and finishers, and substantially in sows (Fig. 3B). Attenuated ATP PRRSV elicited lower levels of anti-nsp2Hp that peaked at 28 days and were maintained for the duration of the study, or declined slightly in piglets (Fig. 3B). Anti-nsp2Hp responses were lowest in sows inoculated with attenuated PRRSV (Fig. 3B), but the same group showed a strong response in HerdChek^® ^PRRS 2XR ELISA (Fig. 2). Thus, pigs of all ages mount a humoral immune response to both virulent and attenuated PRRSV, though its appearance tends to be more rapid in response to virulent virus exposure.

**Figure 4:**
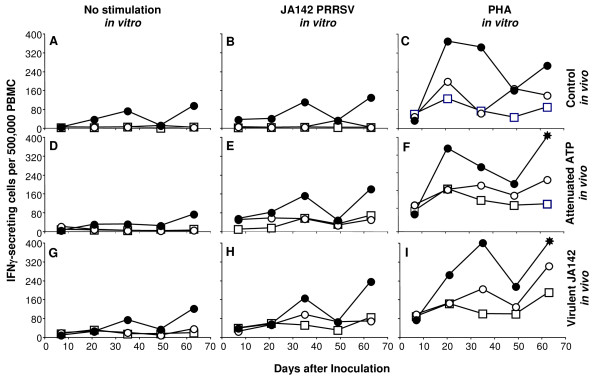
**Effect of pig age and PRRSV strain on interferon (IFN) γ-secreting cell frequencies in peripheral blood mononuclear cells (PBMC)**. Panels A-C, control uninfected pigs (4-5 pigs per group); D-F, pigs inoculated with attenuated ATP PRRSV (5-8 pigs per group); G-I, pigs infected with virulent JA142 (10-11 pigs per group). Panels A, D, and G, PBMC cultured without stimulation; B, E, and H, PBMC cultured with JA142 PRRSV; C, F, and I; PBMC cultured in presence of phytohemagluttinin (PHA). In each panel, piglets are open squares, finisher pigs are open circles, and sows are closed circles. Asterisk in panels F and I indicate wells with too many cells to count, i.e. >400 per well.

Cell-mediated immune responses were examined by IFNγ ELISPOT for evidence that they could explain anti-PRRSV immunity that was not accounted for by antibody responses. Uninfected healthy piglets and finishers showed very low levels of constitutive IFNγ secretion in peripheral blood mononuclear cells (PBMC) alone or after *in vitro *stimulation with virulent PRRSV, whereas mitogenic stimulation increased the frequency of secreting cells (Fig. 4A-C, open circles and open squares). The outcome was similar in piglets and finishers inoculated with attenuated PRRSV, although *in vitro *stimulation with virulent PRRSV increased secreting cell numbers (Fig. 4D-E). PBMC from piglets and finishers infected with virulent PRRSV showed the highest levels of IFNγ secretion under all culture conditions, although there was no consistent change over time (Fig. 4G-I). Sows under all conditions of *in vivo *virus exposure and *in vitro *culture had higher frequencies of IFNγ secreting cells than did piglets and finishers (*p *< 10^-6^, χ^2 ^test). In other respects the trends were the same as in piglets and finishers. Thus, cell-mediated immunity, based on IFNγ secreting cell responses, showed age-dependent variation that was not observed in anti-PRRSV antibody responses.

**Figure 5:**
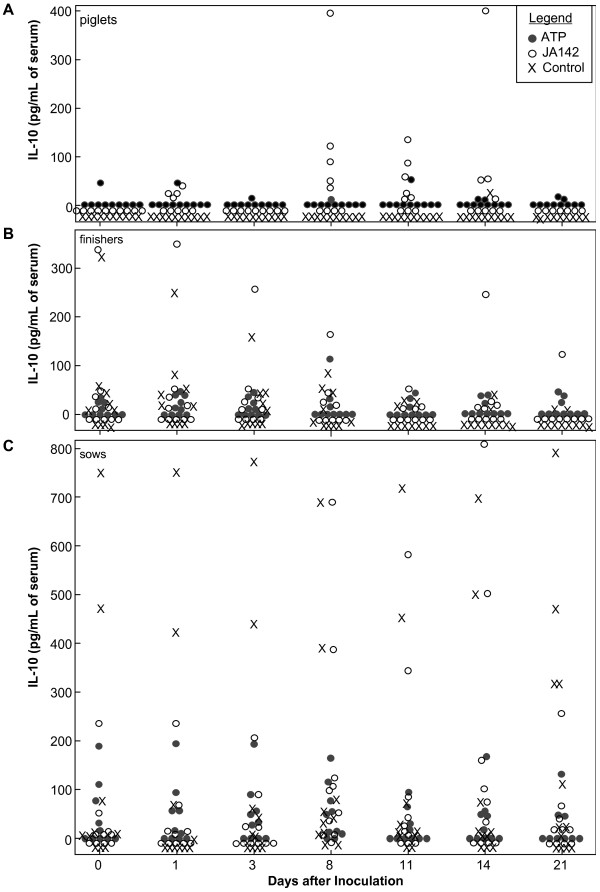
**Effect of pig age and viral strain on IL-10 levels in serum early in infection**. Data points are values from individual piglets (A), finishers (B), and sows (C) treated as indicated in the legend (box in panel A).

The level and duration of viremia were significantly greater in piglets than in finishers and sows. Therefore, we determined IL-10 levels in serum since it has been implicated in delayed immune responses to PRRSV infection [12,13]. In piglets infected with virulent PRRSV, IL-10 levels were significantly increased in serum at 8-14 days of infection (Fig. 5A, p < 0.05). There was no difference between pigs inoculated with attenuated virus or controls. IL-10 levels were more variable in finishers and sows, and there was no difference due to treatment (Fig. 5B, C). In contrast to piglets, approximately half of the finishers and sows before virus exposure had measurable levels of IL-10 that were maintained throughout the study. The results indicate that increased IL-10 levels in serum are associated with age, and that in piglets, increased IL-10 levels are related to viral pathogenesis but do not modulate antiviral immunity.

## Discussion

We show here that the consequences of PRRSV infection are highly dependent on pig age. Viral growth is most extensive in piglets. For both virulent and attenuated PRRSV, peak viremia and duration were substantially greater in piglets. Finishers and sows showed the same pattern of low level viremia for virulent viral infection that resolved within 2 weeks, and approximately 50% of finishers and sows inoculated with attenuated PRRSV showed no viremia. The prolonged period of viremia commonly associated with PRRSV infection is based on studies in young pigs (e.g. [5,14,15]). The finding that viremia is substantially reduced in growing and adult pigs is novel and indicates that the mechanisms of PRRSV resistance are developmentally regulated.

The pattern of slowly rising viral titer, delayed peak and prolonged persistence of viremia observed in piglets given attenuated PRRSV was previously reported in young pigs infected with cell-culture adapted PRRSV strain VR2332 [5]. After one passage *in vivo*, the kinetics of viremia were equivalent to those observed here for infection of piglets with JA142 [5]. Attenuated or lowly virulent PRRSV grow poorly in young pigs [8], but the frequent absence of viremia in older age pigs has not been documented previously.

Age- and viral strain-dependent variation in porcine responses to PRRSV infection was previously reported [9]. However, the significance of the findings in comparison to the present report is uncertain since mature adult pigs were not included, quantitative viral loads were not determined, the viruses were extremely different in genetics as well as virulence, and there was coincident disease in the control group.

The restriction in viral growth in older pigs observed here could be due to differences in innate immunity or in host cell permissiveness. Since the onset of viremia was the same or earlier in finishers and sows compared to piglets, it appears that permissive macrophages are available at all times. Acute infection of pigs at about 20 weeks of age does not reduce the abundance of macrophages in lung or lymphoid tissues [16], therefore it is more likely that suppression of PRRSV infection in older animals is due to more potent mechanisms of innate resistance.

An alternative possibility is that PRRSV selectively induces an immunosuppressive response that blocks innate resistance in young pigs. Various studies based primarily on *in vitro *cell culture experiments that assess mRNA levels or secreted cytokines implicate IL-10 induction by PRRSV infection as a mechanism facilitating viral persistence [13,17,18]. The data here show that IL-10 concentrations are significantly and transiently elevated up to 40 pg/mL in serum of piglets infected with virulent PRRSV. However, older pigs frequently had higher levels of IL-10, up to 800 pg/mL, before infection and in uninfected controls. Since the level of IL-10 prior to infection had no effect on the level of viremia, since peak viremia occurred in piglets one week before the appearance of IL-10, and since it was not observed in piglets exposed to attenuated PRRSV, we conclude that IL-10 production in piglets is a consequence of viral virulence and pathogenesis, rather than being the cause of viral persistence.

In contrast to their effects on infection, pig age and viral virulence had relatively little impact on the antigen-specific adaptive immune response, even though viremia was not observed in nearly half (9/20) of juvenile and adult pigs. Regardless of the viral strain used to challenge pigs, all animals seroconverted, and all groups showed the same level of antibody by HerdChek^® ^PRRS 2XR ELISA at day 35. Variation in the intensity of antibody responses appeared to be random, since differences in kinetics or intensity of response determined by one assay were not reproduced when the same sera were analyzed by another assay, as demonstrated by comparison of the group responses to N and nsp2Hp in Figure 3. Animal variation in the magnitude of immune response to PRRSV and to various PRRSV proteins has been described previously and is a common feature of vertebrate adaptive immunity [8,15,16,19].

Antigen-specific immunological competence is achieved in pigs by day 74 of gestation, i.e. midway in fetal development [20-22]. At 3 weeks of age, piglets show strong IgM and IgG antibody responses to the protein antigen, keyhole limpet hemocyanin, and to a variety of PRRSV proteins following infection [14,15]. Therefore, even if antigen-specific adaptive immunity is not fully developed in piglets, the failure to achieve more rapid elimination of viremia does not appear to be related to the adaptive immune response, as shown in this study and previously [14-16].

Molecular and cellular mechanisms of innate immunity to viral infection are extensive, but little is known about their role in resistance to PRRSV infection. Cellular immunity, mediated by NK cells or other cell types has not been explored [23]. Absence of IFNα induction early in infection is well described and believed to help explain prolonged infection [24-26]. However, there are no comparative studies of differences in interferon responses or other innate immune mechanisms that might explain the marked age-dependent differences in infection outcomes in young versus older pigs. Interleukin-10, which has been suggested to suppress anti-PRRSV immunity [13,17,18], has been shown to suppress inflammatory cytokine production and reduce disease severity in a swine model of bacterial pleuropneumonia [27]. However, the data reported here are more consistent with IL-10 production being a consequence of virulent infection rather than a cause of prolonged infection.

Differences in circulating IFNγ secreting cells did not account for differences in age-dependent infection; rather, they suggested that finishers were more similar to piglets, a conclusion that is in contrast to the similarity between finishers and sows in control of viral infection. The interpretation of IFNγ secreting cell frequencies is confounded since IFNγ in pigs is produced by a wide variety of cell types, including activated CD8+ T cells [28], natural killer T cells [29], and γδT cells [30], in addition to type 1 CD4+ T cells.

The lack of a substantial effect of pig age on antigen-specific immune responses in contrast to a significant age-dependent effect on the kinetics of infection supports the concept that control of PRRSV viremia may not be dependent on adaptive immune responses [14,16,23,31]. Interestingly, lactate dehydrogenase elevating virus, an arterivirus closely related to PRRSV, causes a persistent viremia in mice that is not controlled by a neutralizing antibody response [32]. It has been proposed that infection is controlled, though not eliminated, by a deficiency in permissive macrophages. A similar phenomenon might operate to control PRRSV viremia, which occurs before neutralizing antibody responses are observed [15,18], possibly through interference with virus binding to its CD163 receptor on macrophages [31].

Lastly, it was notable that all groups of pigs exposed to PRRSV developed equivalent adaptive antibody and cell-mediated immune responses irrespective of the kinetics or magnitude of viremia. This observation suggests that the requirements of antigenic mass and mode of presentation for an immune response to PRRSV are met at a low level of infection in the absence of viremia. Therefore, viremia may be an insensitive indicator of infection by lowly virulent or attenuated PRRSV strains, especially in growing and mature swine. Resolution of viremia appears not to require an adaptive immune response based on the findings here and elsewhere [14-16]. While adaptive immunity most likely is essential for protection against future challenge, control of primary infection appears to rely primarily on innate mechanisms of immunity that are more effective at about 15 weeks of age and older.

## Conclusion

We show that animal age, likely due to increased innate immune resistance, strongly influences the outcome of acute PRRSV infection, whereas an effective antibody response is triggered at a low threshold of infection that is independent of age. Prolonged infection was not due to IL-10-mediated immunosuppression, and PRRSV did not elicit a specific IFN γ response, especially in non-adult animals. Equivalent antibody responses were elicited in response to virulent and attenuated viruses, indicating that the antigenic mass necessary for an immune response is produced at a low level of infection, and is not predicted by viremic status. Thus, viral replication was occurring in lung or lymphoid tissues even though viremia was not always observed.

## Methods

### Study Design

Ninety healthy, PRRS-negative pigs, consisting of 30 three-week-old weaned piglets, 30 16-20-week-old mixed-sex finisher pigs, and 30 nonpregnant, third parity (± 1) sows, were obtained from a PRRSV-free, genetically uniform, commercial source herd. Animals were confirmed PRRS-negative by HerdChek^® ^PRRS 2XR ELISA (IDEXX Laboratories Inc., Westbrook, ME) and given a *Mycoplasma hyopneumoniae *vaccine (Boehringer Ingelheim, St. Joseph, MO) on day 0 of the study. Animals were randomized by weight, within each age group, into 3 groups of 10 animals for infection with attenuated Ingelvac^® ^PRRS ATP (Boehringer Ingelheim Vetmedica Inc., St. Joseph, MO), or virulent JA142 PRRSV (vaccine parental isolate, kindly provided by William Mengeling, National Animal Disease Center, Ames, IA) or received diluent only (Table 3). Viral isolates were diluted in Eagle's Minimal Essential Medium (EMEM) (SAFC Biosciences, Lenexa, KS) containing 4% fetal bovine serum (FBS) (SAFC Biosciences, Lenexa, KS) to approximately 3.0 ± 0.5 log_10 _TCID_50_/mL, as determined by titration on MA-104 cells [33]. Treatments were administered as a 1 mL intranasal inoculation and a 1 mL intramuscular injection. As the experiment was not a vaccine evaluation study, the Ingelvac^® ^ATP virus was not taken from a vaccine formulation and the dose and route did not follow USDA-approved label recommendations.

**Table 3:** Experimental study design.

**Group**	**Age**	**Treatment**	**Sample size**	**Observations**
1	Piglet	Ingelvac^® ^PRRS ATP	10	Clinical health, rectal temperature daily. Blood and serum weekly. Lung lesions and tissue samples at necropsy.
2	Finisher	Ingelvac^® ^PRRS ATP	10	Same as above
3	Sow	Ingelvac^® ^PRRS ATP	10	Same as above
4	Piglet	Virulent PRRSV JA142	10	Same as above
5	Finisher	Virulent PRRSV JA142	10	Same as above
6	Sow	Virulent PRRSV JA142	10	Same as above
7	Piglet	Culture media	10	Same as above
8	Finisher	Culture media	10	Same as above
9	Sow	Culture media	10	Same as above

All animals were bled using Vacutainer^® ^serum separation tubes (BD Biosciences, Franklin Lakes, NJ). Serum samples were aliquoted and stored at -70°C until use.

### Viremia quantification

Ten-fold serial dilutions were carried out to a final dilution of 10^-7 ^and four replicates of each dilution were plated on 96-well plates containing three-day-old MA-104 cells. After incubation at 37°C with 4.5% CO_2 _for eight days, wells were examined microscopically for cytopathic effect (CPE). Titer was determined as described [33].

RNA extractions and qRT-PCR were performed as described [8]. Briefly, RNA was isolated by spin-column chromatography (QIAamp Viral RNA Mini-Kit, Qiagen Inc., Valencia, CA) and qRT-PCR was performed using a kit for quantitative detection of PRRSV in serum (Tetracore Inc., Gaithersburg, MD). Results were reported as viral genome copies per mL.

### Serological assays

Seroconversion was quantified as S/P ratios using the HerdChek^® ^PRRS 2XR ELISA according to the manufacturer's instructions. Protein-specific ELISA was performed as described [19,34]. Interleukin-10 levels were determined with a commercial ELISA kit (Biosource International, Camarillo, CA) following the manufacturer's instructions.

### Cell-mediated immune assay

Interferon γ secreting cells were enumerated in PBMC by ELISPOT as described (Xiao et al. 2004). PBMC were cultured at 5 × 10^5 ^cells per well and were stimulated with PRRSV strain JA142 at 2 × 10^5 ^TCID_50 _per well.

### Body weight

Each pig was weighed on days 0 and 63 of the study, using a calibrated, portable, electronic weigh-bar scale (Weigh-Tronix™ model 615XL, Weigh-Tronix Inc., Fairmont, MN). Three-week-old piglets and finishers were also weighed on day 28.

### Clinical scores

Animals were observed daily for clinical condition. Individual scores for respiratory signs, coughing, and behavior were recorded on a scale from 1 (healthy) to 4 (most ill). A healthy pig received a daily score of 3, whereas a dead pig scored a 12. Animals that died prior to the end of the study were necropsied, evaluated for cause of death, and had samples collected for submission to the Iowa State University Diagnostic Lab for confirmation via pathological investigations.

### Statistical analyses

Group mean data for TCID_50_, qRT-PCR, and IDEXX ELISA results was analyzed among age groups, and treatment type for statistical significance using the Kruskal-Wallis non-parametric ANOVA and individual comparisons were analyzed by the Wilcoxon two-sample t-test. Spearman coefficient correlation was used to compare the TCID_50 _and qRT-PCR parameters. A *p *value < 0.05 was considered as statistically significant.

## Competing interests

KLK, MBR, EMV and EMB are employees of Boehringer Ingelheim Vetmedica, which produces a PRRSV vaccine licensed in the USA in which Ingelvac^® ^ATP is the active ingredient. MPM has received unrestricted funds for PRRS and swine immunology research from Boehringer Ingelheim in the previous five years.

## Authors' contributions

KLK carried out the virological and immunological assays and drafted the manuscript. EMV was responsible for the study design, data analysis and manuscript preparation. MBR participated in the study design and performance of the experiment, and interpretation of data. EMB designed and carried out the IL-10 assays, reviewed and edited the manuscript. MPM helped to conceive the study, directed ELISA and ELISPOT assays, and helped write the manuscript. All authors read and approved the final manuscript.
